# Safety and efficacy of CT-guided radioactive iodine-125 seed implantation as a salvage treatment for recurrent head and neck cancer after two or more courses of radiotherapy

**DOI:** 10.1186/s13014-023-02254-z

**Published:** 2023-05-03

**Authors:** Yue Li, Yuliang Jiang, Junjie Wang

**Affiliations:** Department of Radiation Oncology, Peking University Third Hospital, 49 North Garden Road, Haidian District, Beijing, 100191 China

**Keywords:** Head and neck cancer, Radioactive iodine-125 seed implantation, Safety, Efficacy

## Abstract

**Background:**

In the past, patients with recurrent head and neck cancer (rHNC) who had previously received a high dose of radiation and were unable to undergo surgery were mainly treated with palliative chemotherapy due to the high incidence of side effects from re-irradiation. With the development of radiotherapy technology, re-irradiation of recurrent lesions by radioactive iodine-125 seed implantation (RISI) has been proposed as a feasible therapeutic approach. This study aimed to investigate the safety and efficacy of computed tomography (CT)-guided RISI in the treatment of rHNC after two or more courses of radiotherapy, and to analyze the prognostic factors.

**Methods:**

Data of 33 patients with rHNC who received CT-guided RISI after two or more courses of radiotherapy were collected and statistically analyzed. The median cumulative dose of the previous radiotherapy was 110 Gy. Short-term efficacy was assessed by Response Evaluation Criteria in Solid Tumors (version 1.1) criteria, while adverse events were evaluated by Common Terminology Criteria for Adverse Events (version 5.0) criteria.

**Results:**

The median gross tumor volume (GTV) was 29.5 cc, and the postoperative median dose to 90% of target volume (D90) was 136.8 Gy. For adverse reactions, enhanced pain was found in 3 (9.1%) patients, followed by grade 1 to 2 acute skin reactions in 3 (9.1%) patients, grade 2 to 3 late skin reactions in 2 (6.1%) patients, grade 1 to 2 early mucosal reactions in 4 (12.1%) patients, and mandibular osteonecrosis in 1 (3.0%) patient. Regarding the treatment efficacy, the 1- and 2-year local control (LC) rates were 47.8% and 36.4% (median LC time, 10 months), and the 1- and 2-year overall survival (OS) rates were 41.3% and 32.2% (median OS time, 8 months). The absence of adverse events was associated with better LC.

**Conclusions:**

CT-guided RISI, as a salvage therapy, demonstrated acceptable safety and efficacy in the treatment of rHNC after two or more courses of radiotherapy.

**Trial registration:**

This study was registered at Chinese Clinical Trial Register database (Registration No. ChiCTR2200063261) in September 2, 2022.

## Background

Despite the advancement of modern head and neck cancer (HNC) treatment, cancer recurrence is still a major clinical challenge worldwide [1], with a locoregional recurrence rate of 15–50% [2–6]. In the past, patients with recurrent head and neck cancer (rHNC) who had previously received a high dose of radiation and were unable to undergo surgery were mainly treated with palliative chemotherapy due to the high incidence of side effects from re-irradiation. Re-irradiation with three-dimensional conformal radiotherapy (3D-CRT) or intensity-modulated radiation therapy (IMRT) resulted in 10–40% grade 3 and 4 toxicity rates [7]. With the development of radiotherapy technology, re-irradiation of recurrent lesions has been proposed as a feasible therapeutic approach. Low-dose-rate brachytherapy (LDR-BRT) is one of the radiation methods for the treatment of diverse types of cancer, and it has shown advantages for the treatment of recurrent tumors in previously irradiated areas. As a representative technique for LDR-BRT, computed tomography (CT)-guided radioactive iodine-125 seed implantation (RISI) has noticeably attracted clinicians’ attention [8].

The CT-guided RISI may be an appropriate option for patients who cannot receive external beam radiotherapy (EBRT) because of the upper limit of radiation dose for normal tissues. Although most patients in current studies received EBRT before seed implantation, no research has specifically investigated the safety and efficacy of RISI in patients who had previously received two or more courses of radiotherapy.

The present study aimed to investigate side effects and efficacy of CT-guided RISI in the treatment of patients with rHNC after two or more courses of radiotherapy and to analyze prognostic factors.

## Materials and methods

### Patients’ information and selection

We performed a retrospective analysis of patients with rHNC after two or more courses of radiotherapy who underwent CT-guided RISI at Peking University Third Hospital (Beijing, China) from June 2007 to January 2020. The inclusion criteria were as follows: aged ≥ 18 years; patients with rHNC who had previously received two or more courses of EBRT; patients who were pathologically diagnosed with rHNC; no bleeding, no serious or uncontrolled underlying diseases; the availability of appropriate puncture routes, and the expected therapeutic dose could be reached; Karnofsky Performance Status (KPS) score ≥ 60, and tolerant to puncture/radioactive iodine-125 seed implantation, with the expected survival of longer than 3 months. The exclusion criteria were as follows: subjects with skin infection at the puncture site; the lesion was close to the important organs or had a large range of liquefactive necrosis and the seed distribution was poor; subjects who were pregnant, lactating, or mentally ill; poor compliance; ineligible for participating in the clinical trial according to the clinicians’ decision. The study protocol was approved by the Ethics Committee of Peking University Third Hospital (IRB00006761-M2022467), and this study was registered at Chinese Clinical Trial Register database (Registration No. ChiCTR2200063261).

Medical history and physical conditions of all patients were preoperatively assessed, hematological and chemical examinations were performed, and head and neck CT images were obtained. Patients’ characteristics were shown in Table 1. There were 22 male and 11 female patients enrolled in the study. Pathologic examination showed squamous cell carcinoma (25 patients, 75.8%), soft tissue sarcomas (5 patients, 15.2%), malignant melanoma (1 patient, 3.0%), myoepithelial cancer (1 patient, 3.0%), and thyroid carcinoma (1 patient, 3.0%). All patients were considered ineligible for salvage surgery or refused EBRT and surgery.

**Table 1:** Patients’ characteristics (n = 33)

	No. of patients	Percentage (%)
Median age (years)	53(32–72)	
Gender		
Male	22	66.7
Female	11	33.3
Primary tumor position		
Nasopharynx	12	36.4
Hypopharynx	5	15.2
Paranasal sinus	5	15.2
Oral cavity	3	9.1
Larynx	3	9.1
Neck	3	9.1
Cervical lymph node metastases	2	6.1
Tumor pathology		
SCC	25	75.8
Other	8	24.2
Implantation site		
Primary tumor	22	66.7
Lymph nodes	11	33.3
Previous surgery	23	69.7
Previous chemotherapy	20	60.6
Courses of previous radiotherapy		
Two	29	87.9
Three	4	12.1
Cumulative dose of the previous radiotherapy (Gy)		
50–100	11	40.7
> 100	16	59.3
Median dose	110(60–160)	
Median time from radiotherapy to seed implantation (months)	12.5(1.3–446)	

Abbreviations: SCC: Squamous cell carcinoma

## Treatment

### Preoperative planning

**Fig. 1:**
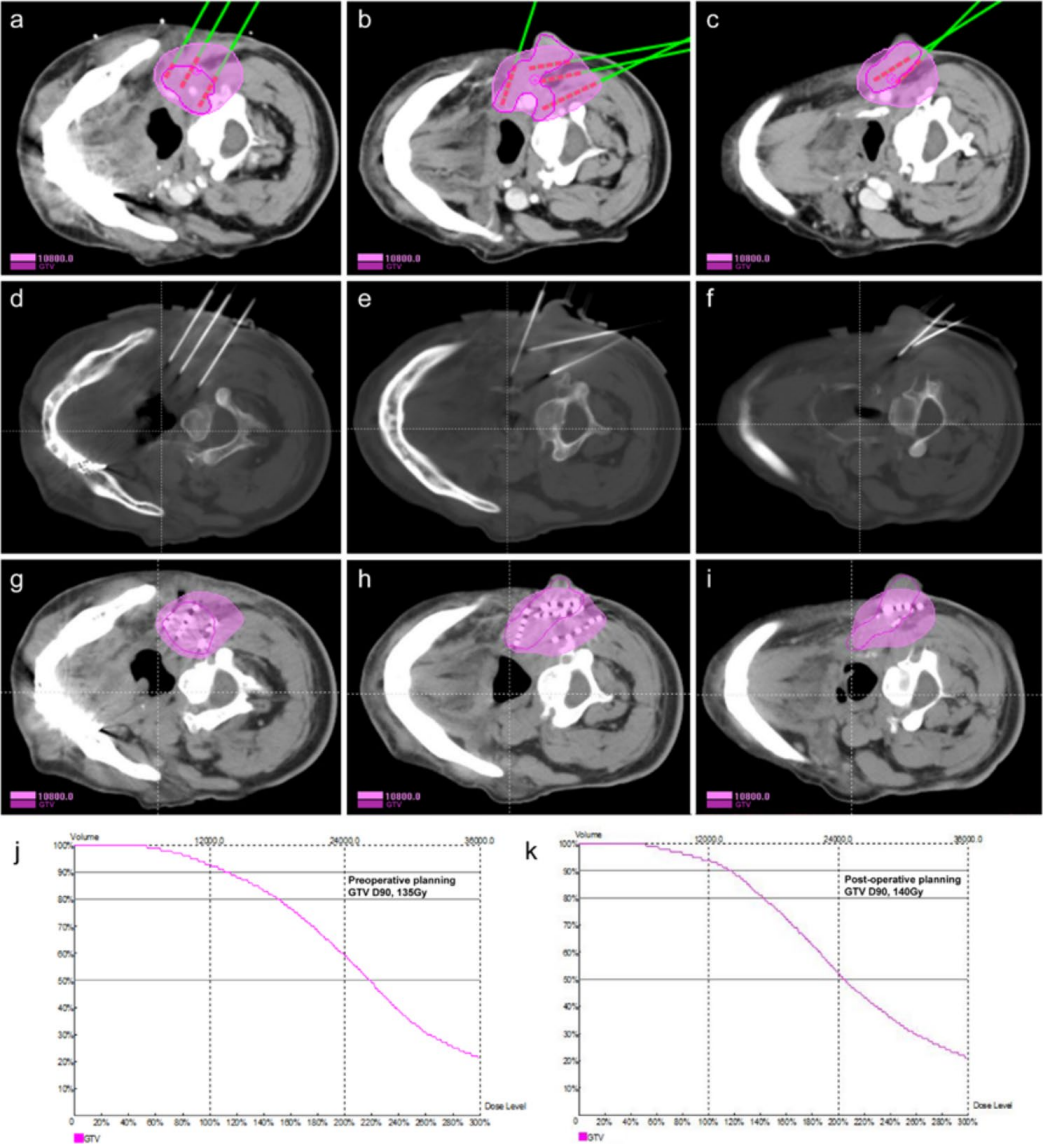
The CT-guided radioactive iodine-125 seed implantation procedure and dose-volume histograms of gross tumor volume Figure **(a-c)** showed the preoperative treatment plan including the planned needle locations, seed distribution, and target volume dose in a case of squamous cell carcinoma of the tongue with cervical lymph node metastasis. The green needles and red seeds were the simulated needles and seeds in the brachytherapy treatment planning system. Figure **(d-f)** showed the actual locations of the needles before the implanting of seeds during operation. Figure **(g-i)** showed the actual distribution of seeds and the dose in target volume after seed implantation. Figure **(j-k)** showed the dose-volume histograms of gross tumor volume preoperation and postoperation

All patients underwent CT scan (slice thickness, 5 mm) during one week before treatment. The corresponding position (supine/prone/side-lying) was selected according to the lesion site, the vacuum pad was fixed, and the body surface was marked with a positioning line. CT data were transmitted to the brachytherapy planning system (BTPS) for preoperative planning. The clinician outlined gross target volume (GTV) and organs at risk (OARs), and set the prescribed dose (110–160 Gy) and radioactive iodine-125 seed activity (0.4–0.8 mCi), determined the seed needle distribution and depth, calculated the number of seeds, and simulated the spatial distribution of seeds. Through software-based calculation and optimization, GTV D90 (dose to 90% of target volume) could reach the prescribed dose. The process of CT-guided RISI was shown in Fig. 1.

### Intraoperative procedures

Under adequate local anesthesia and with reference to the preoperative planning, disposable needles were inserted into the target lesion under CT guidance (at least 0.5 cm from the edge of the tumor on CT, and 0.5-1.0 cm between two rows of needles). Seeds were implanted using a Mick seed implantation gun with a distance of 0.5-1.0 cm between seeds during the withdrawal of the gun. After seed implantation, CT was performed to check the distribution of seeds and additional seeds were implanted to ensure uniform spatial distribution of seeds and to minimize any missed areas.

Since 2016, our department has performed CT-guided RISI with the assistance of three-dimensional printing non-co-planar template (3D-PNCT). After the brachytherapy treatment plan was completed, a digital personal template model was designed in BTPS, including the information of needle distribution, needle path direction, and the features of therapeutic area surface. The 3D-PNCT was then printed by a three-dimensional light-cured rapid-forming printer. Prior to puncture, the 3D-PNCT was aligned to the surface of the treatment area through the outline characteristics of patient, the positioning line on patient, the alignment reference line on 3D-PNCT, and the positioning laser. The implantation needles were then punctured percutaneously to the predetermined depth through guide holes on 3D-PNCT, and seed implantation was performed after the needles’ puncture.

### Postoperative treatment and dose validation

After the surgery, CT scan of the head and neck was performed within 24 h to check any seed displacement and to verify the dose.

### Outcome measures

#### Short-term efficacy

CT was performed to determine changes in tumor size at 3 months after RISI. Treatment response was graded according to the Response Evaluation Criteria in Solid Tumors (version 1.1) criteria, that’s complete response (CR; the disappearance of the target lesion), partial response (PR; at least 30% reduction in the target lesion volume from baseline), progressive disease (PD; at least 20% increase in the target lesion volume), and stable disease (SD; intermediate between PR and PD) [9]. The patients were followed up every 3 months in the first year and every 6 months thereafter.

#### Toxicity and side effects

Adverse events were assessed according to the Common Terminology Criteria for Adverse Events (version 5.0) after seed implantation.

#### Prognostic factors

Factors analyzed were gender, age, pathologic type, number of prior radiotherapy courses, cumulative dose of the previous radiotherapy, previous surgery, previous chemotherapy, implantation site, GTV D90, time from the last external irradiation to seed implantation, short-term efficacy, and adverse effects.

### Statistical analysis

SPSS 23.0 software (IBM, Armonk, NY, USA) was used for statistical analysis. The Kaplan-Meier method was used to calculate local control, survival, and follow-up. The Chi-squared test was employed to analyze the correlation among categorical variables. The log-rank test was utilized for univariate analysis, and Cox regression test was used for multivariate analysis. *P* < 0.05 was considered statistically significant.

## Results

### Seed implantation

Seeds were implanted at the primary tumor site (22 patients, 66.7%) or metastatic lymph nodes (11 patients, 33.3%). The median GTV was 29.5 (1.5-137.7) cc, and the time from radiation therapy to seed implantation was 1.3 to 446 (median, 12.5) months. The number of seeds was 6–121 (median, 51), seed activity ranged from 0.44 to 0.78 (median, 0.58) mCi, and postoperative D90 was 67–246 (median, 136.8) Gy.

### Toxicity and side effects

All patients were followed up for 2 to 36 (median, 27) months. Among 33 patients, a total of 11 patients experienced adverse reactions, and the overall incidence of adverse reactions was 33.3%. All adverse events identified in the study were listed in Table 2.

**Table 2:** Prevalence of side effects in rHNC patients undergoing CT-guided RISI after two or more courses of radiotherapy (n = 33)

	Cases	Percentage
Puncture-related adverse reaction		
Bleeding	0	
Enhanced pain	3	9.1%
Infection	0	
Skin non-union	0	
Implantation metastasis	0	
Radiation-related adverse reaction		
Early skin reaction		
I	1	3.0%
II	2	6.1%
III	0	
IV	0	
Late skin reaction		
I	0	
II	1	3.0%
III	1	3.0%
IV	0	
Early mucosal reaction		
I	1	3.0%
II	3	9.1%
III	0	
IV	0	
Late mucosal reaction	0	
Blood toxicity	0	
Enhanced xerostomia	0	
Radiation myelitis	0	
Radiation-based nerve injury	0	
Mandibular osteonecrosis	1	3.0%
Seed migration	0	

For puncture-related adverse reactions, enhanced pain was found in 3 (9.1%) patients who underwent CT-guided RISI. No case developed an acute reaction of grade ≥ 3. Grade 2 early skin reactions occurred in 2 (6.1%) patients, of whom 1 patient also experienced grade 2 mucosal reactions. Blood toxicity was not observed.

Regarding late reactions, no nerve injury or mucosal reaction occurred. Mandibular osteonecrosis was found in a male patient with grade 3 late skin reaction who received left upper neck metastatic lymph node RISI assisted by 3D-PNCT. The patient’s cervical lymph nodes had previously received two courses of EBRT at a cumulative dose of 136 Gy. Before undergoing CT-guided RISI, this patient developed a significant skin reaction after EBRT. In the RISI treatment, GTV was 4.7 cc, the time from radiation therapy to seed implantation was 18 months, and the postoperative D90 was 136.8 Gy. Three months after seed implantation, his CT examination showed local fibrotic changes in the lymph node without an obvious tumor. However, the patient subsequently developed mandibular osteonecrosis, accompanied by the formation of adjacent skin sinus and intermittent discharge of necrotic tissues. It was speculated that skin and mandibular reactions of the mentioned patient were related to the accumulated dose of irradiation. However, the possibility of tumor invasion could not be excluded.

### Treatment efficacy

**Fig. 2:**
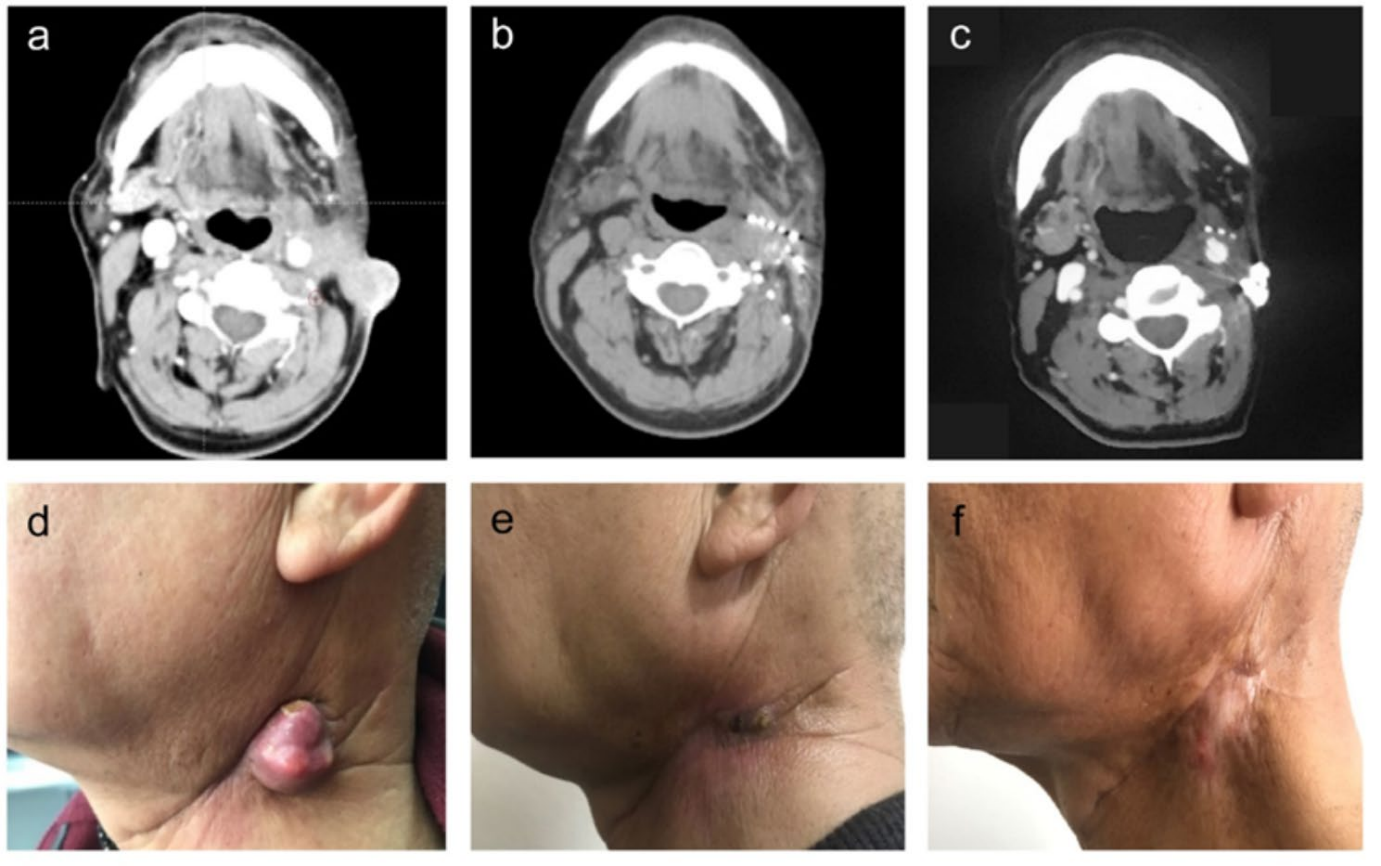
The therapeutic efficacy of CT-guided radioactive iodine-125 seed implantation Figure **(a-c)** showed CT images of the tumor in preoperation, 3-month postoperation, and 18-month postoperation in a case of squamous cell carcinoma of the tongue with cervical lymph node metastasis. Figure **(d-f)** showed the tumors of preoperation, 3-month postoperation, and 18-month postoperation by gross photos, respectively

**Fig. 3:**
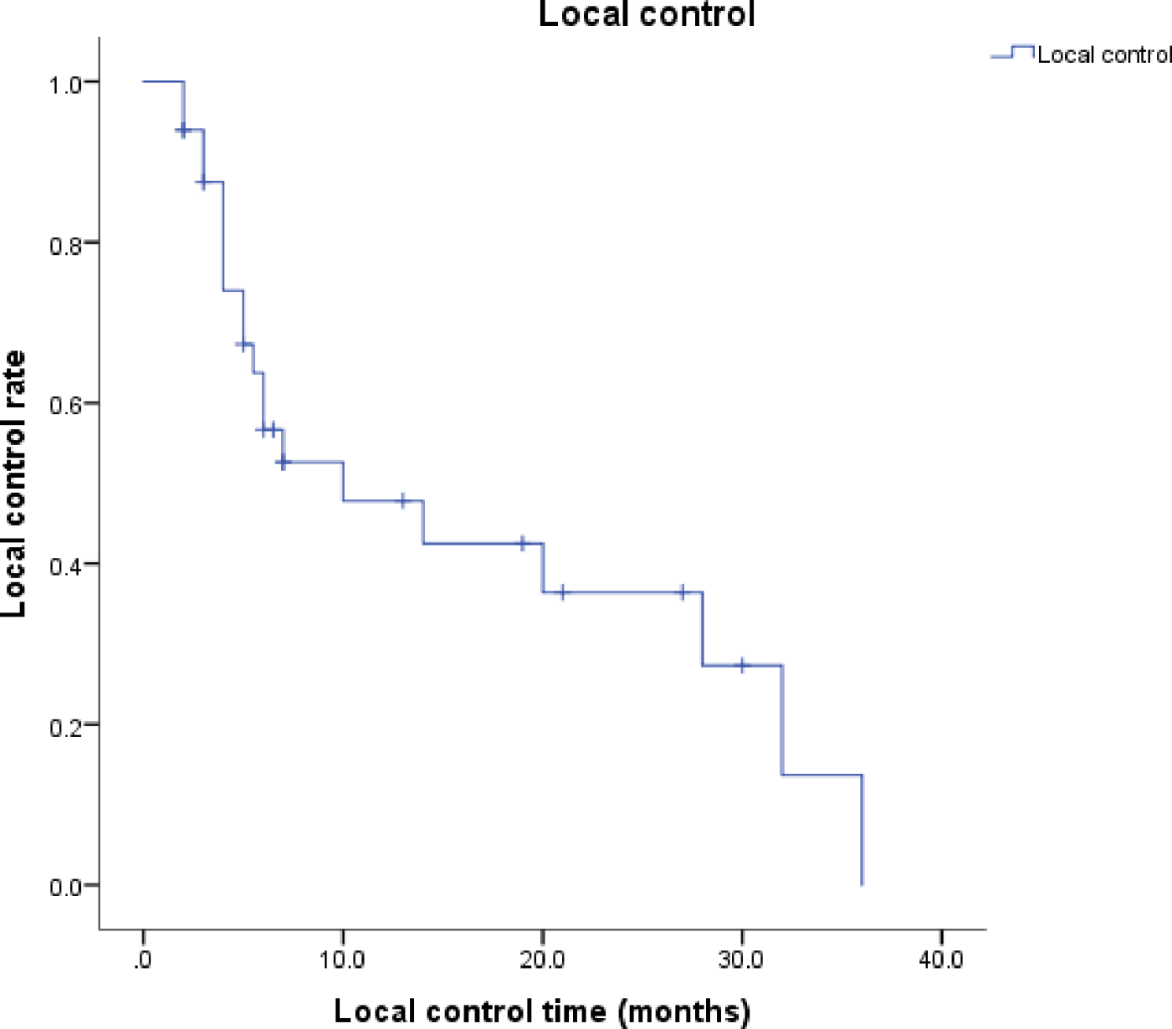
Kaplan–Meier analysis showed local control of 33 patients undergoing CT-guided RISI

**Fig. 4:**
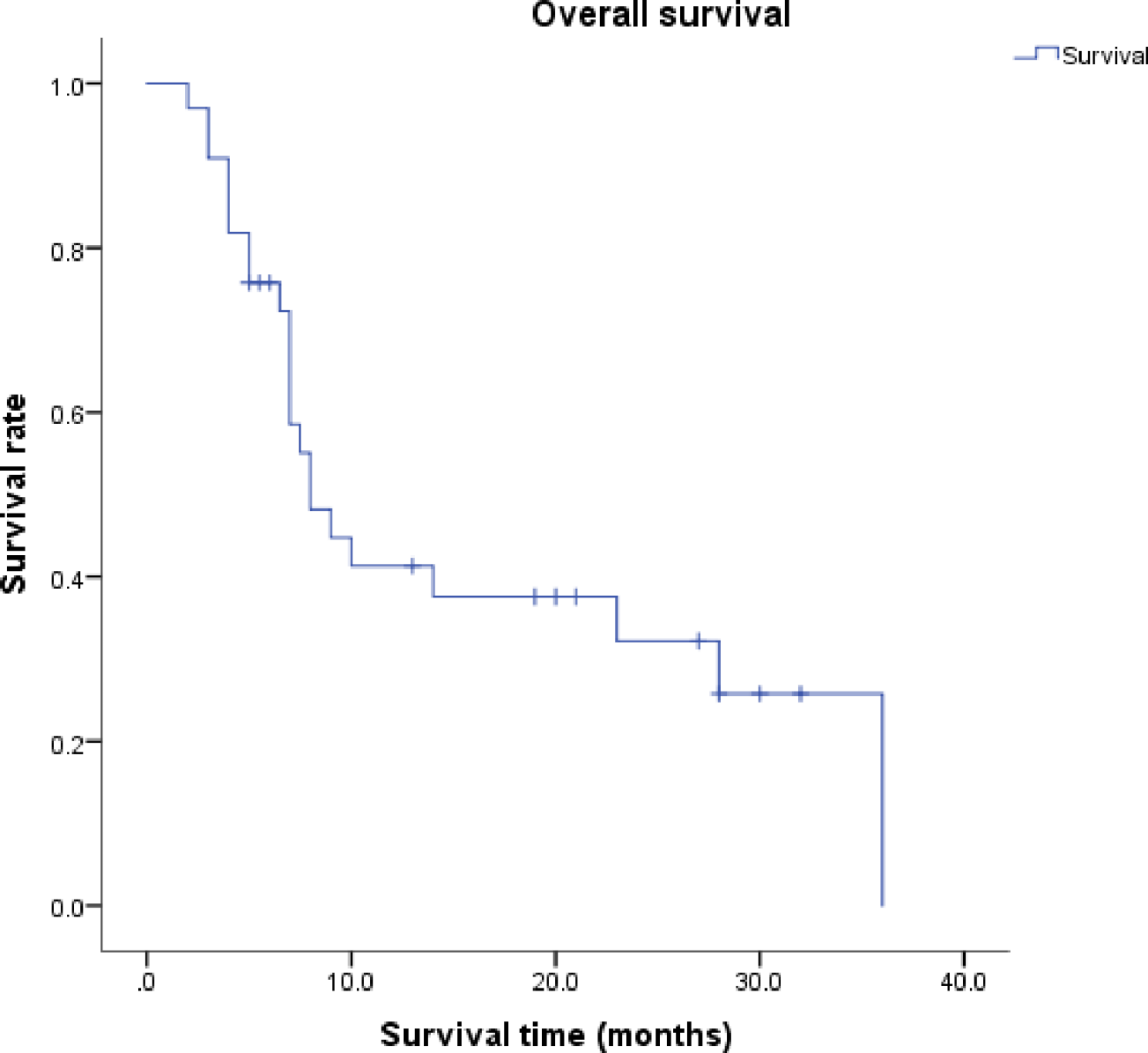
Kaplan–Meier analysis showed overall survival of 33 patients undergoing CT-guided RISI

For the short-term efficacy, 6 (18.2%) patients achieved CR, 16 (48.5%) patients achieved PR, 8 (24.2%) were of SD, and 3 (3.1%) were of PD. The therapeutic effect of RISI in a case was shown in Fig. 2. Local failure occurred in 20 patients (60.6%) within the follow-up period. The median local control (LC) time was 10 months (95% confidence interval (CI), 0-20.671); the 1- and 2-year LC rates were 47.8% and 36.4% (Fig. 3). The median overall survival (OS) time was 8 months (95% CI, 5.409–10.591); and the 1- and 2-year OS rates were 41.3% and 32.2% (Fig. 4).

### Prognostic factors

**Fig. 5:**
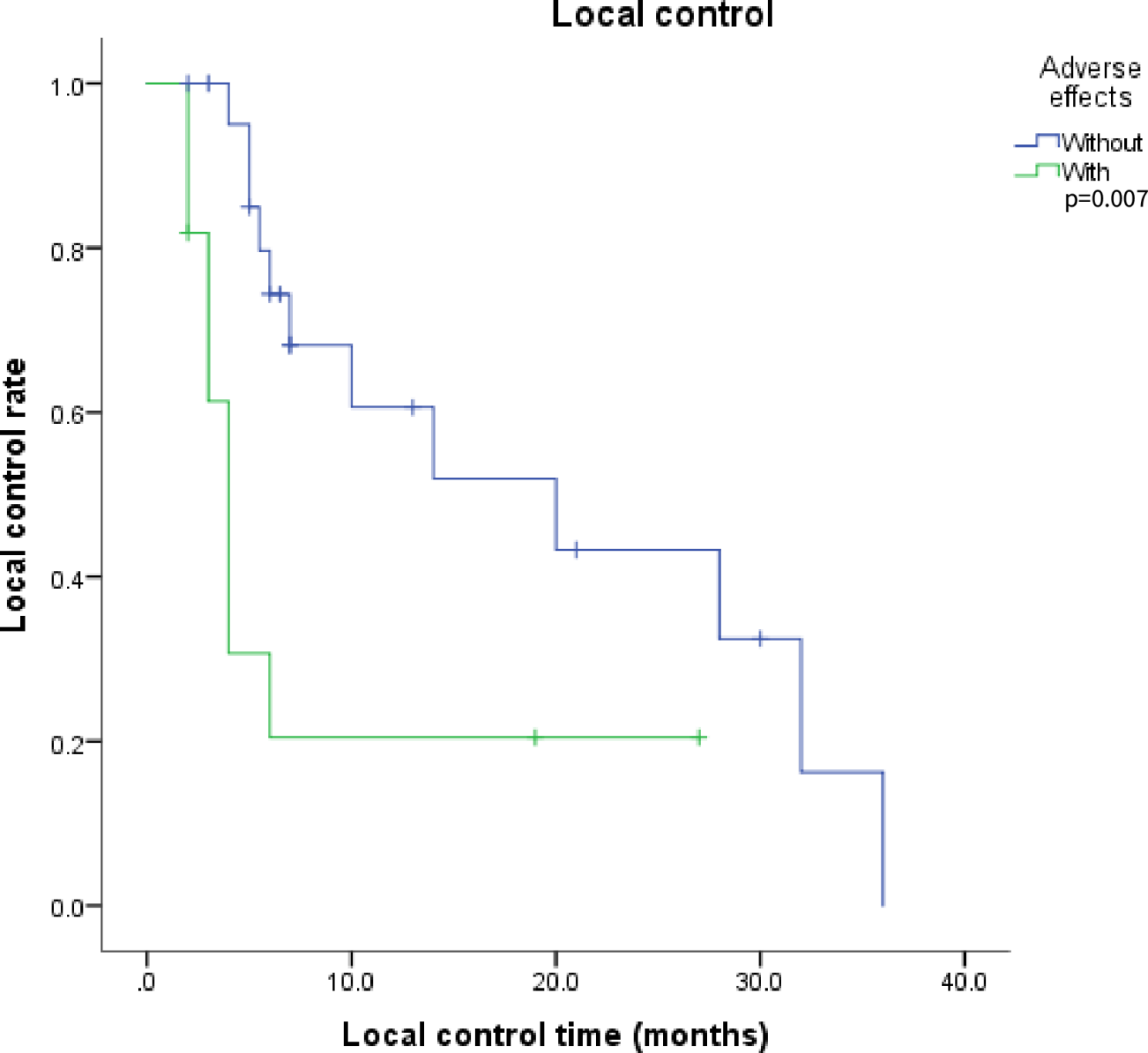
Kaplan–Meier analysis showed a higher LC rate for those without adverse effects compared with those with adverse effects

In this study, univariate analysis showed that the absence of adverse effects was a positive factor for LC (Fig. 5, Chi-squared test: 7.389, *P* = 0.007). No correlation was found among gender, age, pathologic type, number of prior radiotherapy courses, cumulative dose of the previous radiotherapy, previous surgery, previous chemotherapy, implantation site, GTV D90, time from radiation therapy to seed implantation, objective response rate (ORR), and LC or OS (*P* ≥ 0.05). Furthermore, D90 ≥ 135 Gy was associated with the occurrence of adverse effects (Chi-squared test: 6.066, *P* = 0.014). However, there was no association between the cumulative dose of the previous radiotherapy (≥ 110 Gy vs. <110 Gy) and adverse effects (Chi-squared test: 0.297, *P* = 0.586). Multivariate analysis revealed that adverse effects were an independent factor for the LC rate (95% CI: 1.304–9.060, *P* = 0.013), while D90 was not found as a prognostic factor (95% CI: 0.989–1.016, *P* = 0.731).

## Discussion

HNC is the seventh most common type of cancer worldwide, which is typically diagnosed in elderly patients associated with a large amount of tobacco and alcohol use [10]. The majority of HNC patients die of local or regionally persistent or recurrent disease, thus, finding efficacious treatment regimens that can enhance LC of tumors is of great importance [11, 12]. Salvage surgery may be a curative option for patients with resectable locoregional recurrence [13]. However, only 15–30% of patients are eligible for surgery and the 5-year survival rate is 16–36% [14]. Palliative chemotherapy was previously reported as the main choice for patients with inoperable rHNC who received high-dose radiotherapy. Furthermore, the recurrence after multi-course radiotherapy increased the difficulty of reoperation and re-irradiation. With the emergence of new technologies, the re-irradiation of recurrent tumors has markedly attracted clinicians’ attention [7]. Considering the high incidence of re-irradiation toxicities [15, 16], for rHNC, especially for cases who have previously undergone a high cumulative dose of irradiation, it is highly necessary to adopt a radiotherapy regimen with a high conformability and an accurate dose distribution, in order to reduce adverse reactions to normal tissues.

Brachytherapy refers to the use of radionuclides to treat malignant tumors or benign diseases through radioactive sources placed close to or into the tumor or treatment site [17]. Among them, LDR-BRT is achieved by a radioactive source permanently placed in tumor tissues, and the representative technology is RISI.

RISI is guided by imaging, and CT guidance has been widely used as the main imaging technology for seed implantation. After the implantation of radioactive iodine-125 seeds, radiation is continuously released by the radionuclide to achieve the purpose of killing tumor cells [18]. RISI delivers targeted high doses of radiation to tumor tissues with a rapid drop in radiation outside the implanted volume, which minimizes damage to peripheral nerves, vascular structures, adjacent skin, and other OARs [19]. This facilitates application of RISI in the salvage treatment of recurrent solid tumors. Previous studies have shown that RISI is effective for the treatment of rHNC. However, no study has concentrated on the safety and efficacy of seed implantation in patients after two or more courses of radiotherapy, particularly for cases with a high cumulative dose of the previous radiotherapy.

During the follow-up of our study, 11 (33.3%) patients experienced adverse reactions, and only 1 (3.0%) patient developed grade 3 adverse reactions, which may be attributed to treatment-related side effects. Moreover, D90 ≥ 135 Gy was associated with adverse effects, suggesting that for patients with a high cumulative dose of the previous radiotherapy, further attention should be paid to radiotherapy target delineation, and the dose distribution of the target volume and OARs. Ji et al. showed that in patients who underwent seed implantation for rHNC from January 2007 to December 2015 at our department, 16 (15.8%) patients developed grade 1 to 2 skin or mucosal toxicity, 8 (7.9%) patients developed grade 3 skin or mucosal toxicity, and 2 (2.0%) patients suffered grade 4 skin or mucosal toxicity. Pain, which was mainly related to skin pain or mucosal reactions, affected 14 (13.9%) patients, and dry mouth was worsened in 2 (2.0%) patients. Additionally, there was no significant correlation between the implantation dose (D90 < 120 vs. D90 ≥ 120 Gy), the cumulative dose of the previous radiotherapy (≤ 66 vs. > 66 Gy), or the time from EBRT to seed implantation (≤ 18 vs. > 18 months), and grade 3 to 4 skin or mucosal reactions [20]. Since 2016, our department has introduced the 3D-PNCT to assist and improve the accuracy of CT-guided RISI into tumors, which reduces the incidence of severe adverse events. In 2018, Jiang et al. reported the side effects of CT-guided RISI for rHNC assisted by 3D-PNCT. Forty-two patients were enrolled in our hospital from January 2016 to October 2016. It was found that 1 (2.4%) case developed enhanced pain, 3 (7.2%) cases developed grade 1 early skin reactions, and 3 (7.2%) cases suffered grade 1 to 2 early mucosal reactions. The severity of the skin reactions was related to the absorbed dose (D0.1 cc, dose to the most exposed 0.1-cc volume). However, there was little evidence indicating that skin edema and fibrosis were associated with a cumulative dose of the previous EBRT. Additionally, one (2.4%) case had radiation-based nerve injury that might be attributed to tumor involvement on nerves [21]. Jiang et al., Meng et al., Zhu et al., and Chen et al. showed no grade ≥ 4 side effects in their studies [19, 22–24]. In conclusion, the side effects in our study are similar to those reported previously.

For the treatment efficacy of RISI, recent studies have shown that the 1- and 2-year LC rates were 40.6 − 75.2% and 27.5 − 49.9%, and the 1- and 2-year OS rates were 42.5 − 70.8% and 18.2 − 39%, respectively. The median LC time was 10–24 months, and the median OS time was 11–28 months [8, 19, 20, 22–26]. In our study, the median LC time was 10 months; the 1- and 2-year LC rates were 47.8% and 36.4%, respectively. The median survival time was 8 months; and the 1- and 2-year survival rates were 41.3% and 32.2%, respectively. The LC rates in our study were consistent with those reported previously on seed implantation after external irradiation, and the OS rate was slightly lower due to patients’ poor general conditions, indicating that RISI is still an effective treatment plan for patients who mainly cannot receive EBRT again after two or more courses of irradiation. Furthermore, 20 patients (60.6%) experienced local failure during the follow-up period of this study. Because the classification of local failure patterns can better optimize target volume delineation and dose distribution, we will focus on local failure patterns to improve treatment regimens in future studies.

Regarding prognostic factors of seed implantation, our study showed that the absence of adverse effects was a positive factor for LC. Ji et al. evaluated the prognostic factors of CT-guided RISI in the treatment of 101 rHNC patients after EBRT in 2019. Non-squamous cell carcinoma, D90 ≥ 120 Gy, lesion volume ≤ 20 cm^3^, and short-term efficacy (CR + PR) were correlated with better LC, and a higher KPS and lesion volume ≤ 20 cm^3^ were independent factors associated with survival [20]. Chen et al. demonstrated that recurrent T stage and histological grade were prognostic factors for local progression-free survival, while histological grade was noted as a predictor of OS in patients with locally recurrent head and neck soft tissue sarcoma (rHNSTS) after surgery and EBRT [24]. Additionally, Jiang et al. concluded that D90 ≥ 130 Gy was a positive prognostic factor for LC, and location of recurrent lesions and time-to-progression (TTP) were prognostic factors for survival of patients implanted with iodine-125 seeds under ultrasound guidance [8]. In our study, due to the limitation of the sample size, no more prognostic factors were found. Further exploration should be carried out in the future to provide more evidence for treatment planning and prognostic evaluation.

## Conclusions

CT-guided RISI is an effective salvage therapy with a low complication rate for patients with rHNC after two or more courses of radiotherapy. Additional large-scale studies are required to further investigate the efficacy and side effects of CT-guided RISI and to facilitate optimization of treatment techniques.

## Data Availability

Not applicable.

## Abbreviations

HNC: Head and neck cancer
rHNC: Recurrent head and neck cancer
RISI: Radioactive iodine-125 seed implantation
CT: Computed tomography
GTV: Gross tumor volume
D90: Dose to 90% of target volume
LC: Local control
OS: Overall survival
3D-CRT: Three-dimensional conformal radiotherapy
IMRT: Intensity-modulated radiation therapy
LDR-BRT: Low-dose-rate brachytherapy
KPS: Karnofsky Performance Status
BTPS: Brachytherapy planning system
OARs: Organs at risk
3D-PNCT: Three-dimensional printing non-co-planar template
CR: Complete response
PR: Partial response
PD: Progressive disease
SD: Stable disease
CI: Confidence interval
ORR: Objective response rate
D0.1cc: Dose to the most exposed 0.1-cc volume
rHNSTS: Recurrent head and neck soft tissue sarcoma
TTP: Time-to-progression
SCC: Squamous cell carcinoma

## References

[1] Rwigema JC, Heron DE, Ferris RL, Andrade RS, Gibson MK, Yang Y (2011). The impact of tumor volume and radiotherapy dose on outcome in previously irradiated recurrent squamous cell carcinoma of the head and neck treated with stereotactic body radiation therapy. Am J Clin Oncol.

[2] Bourhis J, Le Maître A, Baujat B, Audry H, Pignon JP (2007). Individual patients’ data meta-analyses in head and neck cancer. Curr Opin Oncol.

[3] Brockstein B, Haraf DJ, Rademaker AW, Kies MS, Stenson KM, Rosen F (2004). Patterns of failure, prognostic factors and survival in locoregionally advanced head and neck cancer treated with concomitant chemoradiotherapy: a 9-year, 337-patient, multi-institutional experience. Ann Oncol.

[4] Forastiere AA, Goepfert H, Maor M, Pajak TF, Weber R, Morrison W (2003). Concurrent chemotherapy and radiotherapy for organ preservation in advanced laryngeal cancer. N Engl J Med.

[5] Hall SF, Groome PA, Irish J, O’Sullivan B (2008). The natural history of patients with squamous cell carcinoma of the hypopharynx. Laryngoscope.

[6] Alterio D, Marvaso G, Ferrari A, Volpe S, Orecchia R, Jereczek-Fossa BA (2019). Modern radiotherapy for head and neck cancer. Semin Oncol.

[7] Cengiz M, Özyiğit G, Yazici G, Doğan A, Yildiz F, Zorlu F (2011). Salvage reirradiaton with stereotactic body radiotherapy for locally recurrent head-and-neck tumors. Int J Radiat Oncol Biol Phys.

[8] Jiang P, Wang J, Ran W, Jiang Y, Tian S, Sun H (2019). Five-year outcome of ultrasound-guided interstitial permanent (125)I seeds implantation for local head and neck recurrent tumors: a single center retrospective study. J Contemp Brachytherapy.

[9] Eisenhauer EA, Therasse P, Bogaerts J, Schwartz LH, Sargent D, Ford R (2009). New response evaluation criteria in solid tumours: revised RECIST guideline (version 1.1). Eur J Cancer.

[10] Chow LQM (2020). Head and Neck Cancer. N Engl J Med.

[11] Vokes EE, Weichselbaum RR, Lippman SM, Hong WK (1993). Head and neck cancer. N Engl J Med.

[12] Teudt IU, Kovàcs G, Ritter M, Melchert C, Soror T, Wollenberg B (2016). Intensity modulated perioperative HDR brachytherapy for recurrent and/or advanced head and neck metastases. Eur Arch Otorhinolaryngol.

[13] Argiris A, Karamouzis MV, Raben D, Ferris RL (2008). Head and neck cancer. Lancet.

[14] Rudžianskas V, Inčiūra A, Vaitkus S, Padervinskis E, Rudžianskienė M, Kupčinskaitė-Noreikienė R (2014). Reirradiation for patients with recurrence head and neck squamous cell carcinoma: a single-institution comparative study. Med (Kaunas).

[15] Langer CJ, Harris J, Horwitz EM, Nicolaou N, Kies M, Curran W (2007). Phase II study of low-dose paclitaxel and cisplatin in combination with split-course concomitant twice-daily reirradiation in recurrent squamous cell carcinoma of the head and neck: results of Radiation Therapy Oncology Group Protocol 9911. J Clin Oncol.

[16] Roh KW, Jang JS, Kim MS, Sun DI, Kim BS, Jung SL (2009). Fractionated stereotactic radiotherapy as reirradiation for locally recurrent head and neck cancer. Int J Radiat Oncol Biol Phys.

[17] Viswanathan AN, Erickson BA, Ibbott GS, Small W, Eifel PJ (2017). The American College of Radiology and the american Brachytherapy Society practice parameter for the performance of low-dose-rate brachytherapy. Brachytherapy.

[18] Zhang F, Wang J, Guo J, Li Y, Huang X, Guan Z (2018). Chinese Expert Consensus Workshop Report: Guideline for permanent iodine-125 seed implantation of primary and metastatic lung tumors. Thorac Cancer.

[19] Zhu L, Jiang Y, Wang J, Ran W, Yuan H, Liu C (2013). An investigation of 125I seed permanent implantation for recurrent carcinoma in the head and neck after surgery and external beam radiotherapy. World J Surg Oncol.

[20] Ji Z, Jiang Y, Tian S, Guo F, Peng R, Xu F (2019). The effectiveness and prognostic factors of CT-Guided Radioactive I-125 seed implantation for the treatment of recurrent Head and Neck Cancer after External Beam Radiation Therapy. Int J Radiat Oncol Biol Phys.

[21] Jiang Y, Ji Z, Guo F, Peng R, Sun H, Fan J (2018). Side effects of CT-guided implantation of (125)I seeds for recurrent malignant tumors of the head and neck assisted by 3D printing non co-planar template. Radiat Oncol.

[22] Jiang YL, Meng N, Wang JJ, Jiang P, Yuan H, Liu C (2010). CT-guided iodine-125 seed permanent implantation for recurrent head and neck cancers. Radiat Oncol.

[23] Meng N, Jiang YL, Wang JJ, Ran WQ, Yuan HS, Qu A (2012). Permanent implantation of Iodine-125 seeds as a salvage therapy for recurrent Head and Neck Carcinoma after Radiotherapy. Cancer Invest.

[24] Chen Y, Jiang Y, Ji Z, Jiang P, Xu F, Zhang Y (2020). Efficacy and safety of CT-guided (125)I seed implantation as a salvage treatment for locally recurrent head and neck soft tissue sarcoma after surgery and external beam radiotherapy: a 12-year study at a single institution. Brachytherapy.

[25] Jiang YL, Meng N, Wang JJ, Ran WQ, Yuan HS, Qu A (2010). Percutaneous computed tomography/ultrasonography-guided permanent iodine-125 implantation as salvage therapy for recurrent squamous cell cancers of head and neck. Cancer Biol Ther.

[26] Jiang P, Jiang Y, Wang JJ, Meng N, Ran W, Qu A (2011). Percutaneous ultrasonography-guided permanent iodine-125 implantation as salvage therapy for recurrent head and neck carcimonas. Cancer Biother Radiopharm.

